# Socioeconomic correlates of overweight and obesity among ever-married urban women in Bangladesh

**DOI:** 10.1186/s12889-019-7221-3

**Published:** 2019-06-28

**Authors:** Tania Sultana Tanwi, Sayan Chakrabarty, Syed Hasanuzzaman, Sue Saltmarsh, Stephen Winn

**Affiliations:** 1 Maternal and Child Health Division, icddr,b, Mohakhali, Dhaka, 1212 Bangladesh; 2 Department of Economics, Shahjalal University of Science & Technology, Sylhet Kumargaon, Sylhet, 3114 Bangladesh; 3 Faculty of Business, Education, Law and Arts, University of Southern Queensland, Springfield, QLD 4300 Australia; 4 School of Teacher Education and Early Childhood, Faculty of Business, Education, Law and Arts, University of Southern Queensland, Springfield, Australia; 5 School of Education, Edith Cowan University, Joondalup, Australia

**Keywords:** Socioeconomic, Overweight, Obesity, Urban women, Bangladesh

## Abstract

**Background:**

The escalating prevalence of overweight and obesity globally is reflected amongst urban women in many low-to-middle income countries. Evidence also shows that overweight and obesity is an increasing trend in Bangladesh. The present study assessed the prevalence and socioeconomic determinants of overweight and obesity among urban women in Bangladesh.

**Methods:**

Data were extracted from Bangladesh Demographic and Health Survey (BDHS) 2014. A two-stage stratified sampling technique has been used for data collection in this cross-sectional survey. A sample of 1701 ever-married non-pregnant urban women aged 15–49 years was selected for statistical analysis. Descriptive analysis, multiple binomial logistic regression analysis were executed in this study.

**Results:**

The prevalence of overweight and obesity was 34% (95% CI, 0.30–0.38) among urban Bangladeshi women. The probability of being overweight and obese increased with increasing age and wealth index. The likelihood of being overweight and obese among the oldest women surveyed (40–49 years) was 4.3 times (OR = 4.3, 95% CI: 2.1–8.8) higher relative to the youngest women (15–19 years). The wealthiest women had 4.1 times (OR = 4.1, 95% CI: 2.5–6.7) higher likelihood of being overweight and obese compared to the reference group of poorest women. Women having higher education (OR = 1.7, 95% CI: 1.0–2.6) were more likely to be overweight and obese. However, women who were no longer living with their husband or separated from their husband were (OR = 0.4, 95% CI: 0.2–0.8) less likely to be overweight and obese.

**Conclusion:**

This study provides evidence that a large number of urban women were overweight and obese in Bangladesh. Women having higher levels of education, being older and belonging in both poorer and richest wealth quintile were at risk of being overweight and obese. Appropriate health promoting interventions based on these factors should be envisaged to reduce this problem.

## Background

Worldwide, the prevalence of overweight and obesity is rapidly escalating with approximately 641 million obese people throughout the world in 2014 [1]. It has been projected that globally 1.35 billion and 573 million people would be overweight and obese by 2030 [2]. Overweight and obesity has detrimental health outcomes, including a variety of non-communicable diseases (NCDs) [3, 4]. The higher prevalence of overweight and obesity has been found among women than men [5]. Moreover, increases in some forms of cancer such as endometrial cancer, postmenopausal breast cancer and ovarian cancer among women have also been linked to increases in rates of overweight and obesity [6]. While previously overweight and obesity was seen as a problem of high income countries, it is now a problem in many low-and-middle income countries, particularly for those who reside in urban areas [7, 8].

Evidence shows that the prevalence of overweight and obesity is increasing in Bangladesh, especially among women [9–13]. The prevalence of overweight and obesity has been raised from 4 to 16% during the period between 1996 to 2011 [10], according to Bangladesh Demographic and Health Survey (BDHS) [14]. Furthermore, the burden of overweight and obesity has been found to be higher among urban women in Bangladesh compared to their rural counterparts [9], the prevalence among urban women was increased by 17.5% between 1996 and 2011 [10].

Bangladesh is undergoing a rapid urbanization process [15]. A number of studies have identified urbanization as one of the major determinants of increasing overweight and obesity [12, 16] and the prevalence of overweight and obesity is higher in urban areas than rural areas [17–19]. Moreover, women are affected more by the consequences of being overweight and obese compared to men [20]. Additionally, ever-married women are more susceptible to being overweight and obese than never-married women [21]. Little effort has been made to address determinants of overweight and obesity in urban married women. Therefore, this study attempted to investigate socioeconomic and demographic factors of overweight and obesity among ever-married urban women in Bangladesh. Hopefully, this study will enhance information available for future research and contribute to appropriate interventions for combating overweight and obesity in Bangladesh.

## Methods

### Study area

Bangladesh is a South-Asian country with an area of 147,570 km^2^. According to the latest decennial population census by Bangladesh Bureau of Statistics (BBS), the population (adjusted) of the country was estimated at 149.77 million in 2011 of which about 74.98 million was male and 74.79 million was female [22]. The Human Development Index (HDI) value of 0.579 for 2015 designated this country as a medium development category [23]. The United Nations has estimated that approximately 54 million people of this country were urban resident in 2015. Although the majority of the population lives in rural areas, the number of urban dwellers is increasing rapidly. It is projected that majority of the people will be urban residents by 2039 [24]. Urban populations are diverse in terms of various socioeconomic and health related issues [15].

### Study population

The data used in this study were drawn from the most recent 2014 Bangladesh Demographic Health Survey (BDHS) which is the seventh nationally representative demographic and health survey. This survey, implemented by Bangladeshi research organization Mitra and Associates, covered broad areas of information including demographic status, nutritional status, family planning, maternal health and children’s health. Technical support was provided by ICF International of Rockville, Maryland, USA, and financial assistance was provided by the United States Agency for International Development (USAID). A two-stage stratified cluster sampling design based on the 2011 Population and Housing Census of Bangladesh has been used in this nationally representative cross-sectional survey to estimate key indicators for each of the seven administrative divisions in Bangladesh. Details of the survey design, sampling technique, questionnaire and quality control are narrated in the BDHS 2014 report [25]. Anthropometric data (height and weight) were measured by trained personnel using standardized procedures.

**Fig. 1:**
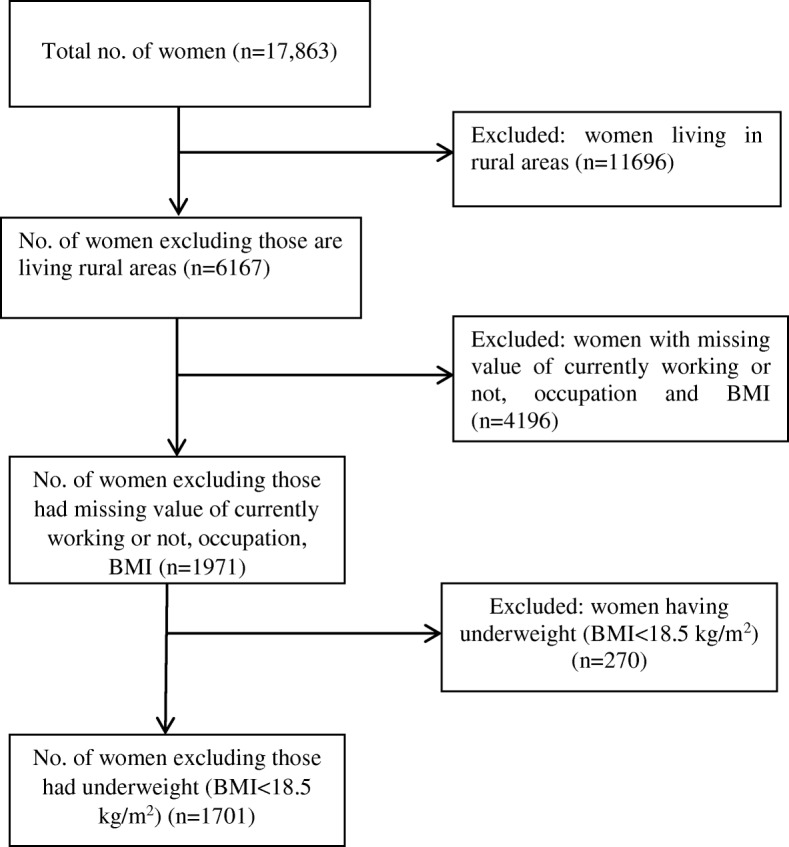
Schematic diagram of data extraction from BDHS 2014

Data have been extracted from the Women’s Questionnaire for this study, with a sample of 17,863 married women. Following procedure has been used for data extraction: data were first extracted from women file BDHS 2014, and only the required variables were subsequently extracted. As our study was restricted to urban women, women living in rural areas were excluded. Data with missing information were also excluded. As this study focused only on overweight and obese women, underweight (BMI < 18.50 kg/m^2^) [26] women were excluded from raw data. Finally, 1701 women were selected for this study. The schematic diagram (Fig. 1) shows the steps of extracting data.

### Variable description

In this analysis, overweight and obesity were considered as outcome variables which were measured by Body Mass Index (BMI). BMI was calculated as: $\mathrm{BMI}=\frac{\mathrm{weight}\kern0.34em \mathrm{in}\kern0.34em \mathrm{kilogram}\kern0.2em \left(\mathrm{kg}\right)}{\mathrm{height}\kern0.34em \mathrm{in}\kern0.34em \mathrm{meter}\kern0.34em \mathrm{squared}\kern0.2em \left({m}^2\right)}$. According to the definition of the World Health Organisation [WHO] [26], overweight was considered where BMI > =25.00 kg/m^2^ and < = 29.99 kg/m^2^ and obesity where BMI > = 30.00 kg/m^2^.

In this analysis, dependent variable was categorized as overweight and obese where respondents’ weight exceeded reference group normal weight (BMI > =18.50 kg/m^2^ and < =24.99 kg/m^2^). Socioeconomic and demographic characteristics of the women were used as predictor variables such as age (in years) (categorized as 15–19, 20–29, 30–39, 40–49), region of residence was categorized by seven administrative divisions in Bangladesh (Barisal, Chittagong, Dhaka, Khulna, Rajshahi, Rangpur, Sylhet). Educational status was stratified by no education, primary education, secondary education and higher education. Marital status was also divided into four categories as married, widowed, divorced and no longer living together/ separated. Parity was grouped by no children, 1–2 children, 3–4 children, 5^+^ children. Household wealth status was measured by wealth index as poorest, poorer, middle, richer, richest. The wealth index was constructed using data on selected household assets by principle component analysis (PCA). Another predictor variable, working status (not working, white-collar, manual, other) was also used. The working status variable was constructed using the information regarding whether the respondent is currently working or not, and the respondent’s occupation.

### Statistical analysis

Analysis of data began with descriptive statistics of the study population such as frequencies and proportion in relation to several selected socioeconomic and demographic variables. Multiple binomial logistic regression analysis was executed to find out association of each predictor variable with outcome variable. Crude odds ratios and adjusted odds ratios with corresponding 95% confidence intervals (CIs) were estimated. *P*-value of 0.05, 0.01 and 0.001 showed statistical significance. The full analysis was done by using statistical software STATA (version 13.0).

## Results

### Characteristics of the sample

In this analysis, urban women aged 30–39 years were 618 (36.3%) among the 1701 urban women included in the sample. Approximately, 487 (28.6%) had primary education and 1501 (88.2%) were married. The majority of the urban women were in richest quintile 640 (37.6%) followed by the richer 517 (30.4%) and women in the middle quintile 261 (15.3%) of wealth index. Almost half of urban women (55.3%) had 1–2 children (Table 1).

**Table 1:** : Characteristics of ever married non pregnant reproductive age women in urban Bangladesh, 2014 BDHS

Variables	Frequency (%)
Age	
15–19	90 (5.3)
20–29	572 (33.6)
30–39	618 (36.3)
40–49	421 (24.8)
Weight (kilogram)^a^	54.4 (9.3)
Height (meter)^a^	15.1 (0.6)
BMI (kg/m^2^)^a^	23.9 (3.6)
Highest Educational Level	
No education	433 (25.5)
Primary	487 (28.6)
Secondary	477 (28.0)
Higher	304 (17.9)
Current Marital Status	
Married	1501 (88.2)
Widowed	92 (5.4)
Divorced	36 (2.1)
No longer living together / separated	72 (4.2)
Parity	
No children	147 (8.6)
1–2 children	941 (55.3)
3–4 children	458 (26.9)
5+ children	155 (9.1)
Working Status	
No working	114 (6.7)
White-collar	353 (20.8)
Manual	1003 (59.0)
Other	231 (13.6)
Wealth Index	
Poorest	166 (9.8)
Poorer	117 (6.9)
Middle	261 (15.3)
Richer	517 (30.4)
Richest	640 (37.6)
Region of Residence	
Barisal	170 (10.0)
Chittagong	262 (15.4)
Dhaka	420 (24.7)
Khulna	272 (16.0)
Rajshahi	256 (15.1)
Rangpur	201 (11.8)
Sylhet	120 (7.1)
Nutritional Status	
Normal	1123 (66.0)
	(95% CI: 0.64–0.68)
Overweight	486 (28.6)
	(95% CI: 0.26–0.31)
Obese	92 (5.4)
	(95% CI: 0.04–0.07)

^a^ notation expressed data as mean (standard deviation)

### Prevalence of overweight and obesity and its socioeconomic determinants

From the descriptive statistics (Table 1), the prevalence of overweight was 28.6% (95% CI: 0.26–0.31) and obesity was 5.4% (95% CI: 0.04–0.07). Overall, 34% (95% CI, 0.30–0.38) urban ever-married reproductive age women were overweight and obese.

Binomial logistic regression analysis was also presented in Table 2. Findings revealed that wealth index, educational status, age and marital status have significant association with overweight and obesity.

**Table 2:** : Socioeconomic determinants of being overweight and obese among women in urban Bangladesh, 2014, BDHS

Variable	Overweight and Obesity		Crude Odds Ratio (95%CI)	Adjusted Odds Ratio (95% CI)
	Yes (%)	No (%)		
Age				
15–19	11 (12.2)	79(87.8)	1	1
20–29	148 (25.9)	424(74.1)	2.5 (1.3–4.8)^a^	2.1 (1.0–4.1)^a^
30–39	249 (40.3)	369 (59.7)	4.8 (2.5–9.3)^c^	3.9 (1.9–7.7)^c^
40–49	170 (40.4)	251 (59.6)	4.9 (2.5–9.4)^c^	4.3 (2.1–8.8)^c^
Highest educational Level				
No education	120(27.7)	313 (72.3)	1	1
Primary	137(28.1)	350 (71.9)	1.0 (0.8–1.4)	1.1 (0.8–1.5)
Secondary	160 (33.5)	317 (66.5)	1.3 (1.0–1.7)	1.2 (0.8–1.6)
Higher	161 (53.0)	143 (47.0)	2.9 (2.2–4.0)^c^	1.7 (1.0–2.6)^a^
Current Marital Status				
Married	532 (35.4)	969 (64.6)	1	1
Widowed	29 (31.5)	63 (68.5)	0.8 (0.5–1.3)	0.7 (0.5–1.2)
Divorced	6 (16.7)	30 (83.3)	0.4 (0.2–0.9)^a^	0.4 (0.2–1.1)
No longer	11 (15.3)	61 (84.7)	0.3 (0.2–0.6)^c^	0.4 (0.2–0.8)^a^
Living together/				
separated				
Parity				
No children	33 (22.5)	114 (77.5)	1	1
1–2 children	340 (36.1)	601 (63.9)	2.0 (1.3–2.9)^c^	1.5 (0.9–2.3)
3–4 children	151 (33.0)	307 (67.0)	1.7 (1.1–2.6)^a^	1.3 (0.8–2.2)
5+ children	54 (34.8)	101 (65.2)	1.8 (1.1–3.1)^a^	1.6 (0.9–3.0)
Working Status				
No working	31 (27.2)	83 (72.8)	1	1
White-collar	164 (46.5)	189 (53.5)	2.3 (1.5–3.7)	1.2 (0.7–2.0)
Manual	327(32.6)	676 (67.4)	1.3 (0.8–2.0)	1.2 (0.8–1.9)
Other	56 (24.2)	175 (75.8)	0.9 (0.5–1.4)	0.8 (0.5–1.4)
Wealth Index				
Poorest	29 (17.5)	137 (82.5)	1	1
Poorer	34 (29.1)	83 (70.9)	1.9 (1.1–3.4)^b^	1.9 (1.1–3.5)^a^
Middle	79 (30.3)	182 (69.7)	2.1 (1.3–3.3)^c^	2.3 (1.4–3.8)^c^
Richer	150 (29.0)	367 (71.0)	1.9 (1.2–3.0)^c^	2.4 (1.5–3.8)^c^
Richest	286 (44.7)	354 (55.3)	3.8 (2.5–5.9)^c^	4.1 (2.5–6.7)^c^
Region of Residence				
Barisal	62 (36.5)	108 (63.5)	1	1
Chittagong	78 (29.8)	184 (70.2)	0.7 (0.5–1.1)	0.8 (0.5–1.2)
Dhaka	130 (30.95)	290 (69.05)	0.8 (0.5–1.1)	0.8 (0.5–1.2)
Khulna	102 (37.5)	170 (62.5)	1.0 (0.7–1.6)	1.2 (0.8–1.8)
Rajshahi	100 (39.1)	156 (60.9)	1.1 (0.7–1.7)	1.4 (0.9–2.1)
Rangpur	74 (36.8)	127 (63.2)	1.0 (0.7–1.6)	1.1 (0.7–1.8)
Sylhet	32 (26.7)	88 (73.3)	0.6 (0.4–1.1)	0.8 (0.4–1.3)

^a^significant at 0.05

^b^significant at 0.01

^c^significant at 0.001

The odds of being overweight and obese were 3.9 times (95% CI: 1.9–7.7) higher among 30–39 years and 4.3 times (95% CI: 2.1–8.8) higher among 40–49 years of age group compared to the women of age group 15–19 years old. Higher educated women (OR = 1.7, 95% CI: 1.0–2.6) were more likely to be overweight and obese compared to women who have no education. The probability of being overweight and obese was 4.1 times (95% CI: 2.5–6.7) higher among richest women relative to the poorest women. Women who are no longer living together with their husbands or separated from their husbands (OR = 0.4, 95% CI: 0.2–0.8) were less likely to be overweight and obese compared to married women.

## Discussion

This study provides evidence that a large number of urban women were overweight and obese in Bangladesh and also identifies several socioeconomic factors that were associated with urban women becoming overweight and obese. Specifically, socioeconomic determinants such as wealth index, age, marital status and educational status were associated with being overweight and obese among urban women in Bangladesh.

The present study shows that the prevalence of overweight and obesity among urban women was 34%, which was higher than the national average (24%). However, it was lower than the estimate of urban women reported by BDHS 2014 [25]. This discrepancy accrued as we chose observations for our analysis on the basis of several selected socioeconomic characteristics. The prevalence of overweight and obesity of this present study was higher (34%) than another study (19.6%) based on BDHS 2011 which indicates increasing prevalence of overweight and obesity among urban women in Bangladesh [12]. Compared to the other studies based on DHS data, the prevalence of overweight and obesity from this study was higher than Ethiopia (14.9%) [27], Nigeria (26.7 and 36.4%) [28] and lower than South Africa (56.6%) [29], Benin (41.3%) [30], Iran (61.3%) [21], India (75.33%) [31]. The consecutive economic development and rapid growth of urbanization in developing countries are positively correlated with the prevalence of being overweight [32]. Access to advanced technologies which help to do work with less energy, consumption of energy-dense food, congested space for physical activity and more sedentary lifestyles have all been shown to contribute to overweight and obesity in urban regions [7, 33, 34]. One study revealed that among South-Asian countries, Bangladesh has the highest levels of physical inactivity and poor dietary habits [35].

The findings of this study include identification of various factors that were associated with urban women in Bangladesh being overweight and obese. The study has identified that the older women surveyed had greater probability of being overweight and obese compared to younger women which is consistent with findings from many other studies [9, 12, 27, 36]. Intake of more energy-dense food as well as having less physical activity may explain why women’s likelihood of being overweight and obese increases with age [37]. The cumulative effect of having a positive energy balance over the life course might be a reason for higher overweight and obesity rates with higher age [38]. Additionally, given that fat mass rises and also fat-free mass declines when a person crosses 30 years of his or her age, a possible explanation may also lie in the association of higher age with changes in body composition [39, 40].

The analysis also reveals that women in the richest quintile were more likely to be overweight and obese compared to poorest women. This is consistent with several similar studies of Bangladesh [9, 10, 12] and elsewhere [17, 27, 36]. Studies in other parts of Asia have shown that the intake of higher fat and consumption of energy dense food products increases with the rise in income, hence dietary factors of this sort may also be a possible reason for the greater likelihood of wealthier women in Bangladesh being overweight and obese [41]. This study also found that poorer women were more likely to be overweight and obese compared to the poorest women. One possible explanation for this might be unhealthy dietary habits among poorer women, leading to a greater likelihood of this group being overweight and obese [42].

Higher educational qualification has strong association with women being overweight and obese, which is also consistent with findings from other similar studies [9, 10, 12, 27]. This includes research finding that highly educated women have a greater likelihood of being obese than less educated women in developing countries [43]. The expected reason for this may be that higher educational levels lead women into more sedentary occupations rather manual labor, resulting in less physical activity. For example, a study in Iran found that higher educational status was negatively related with their obesity in both cases of men and women [44]. Other studies conducted in Bangladesh showed region of residence was significantly associated with overweight and obesity [9, 12]. However, this indicator was found to be insignificant in this study.

### Strengths and limitations

The high prevalence of overweight and obesity among urban women is a great challenge for public health globally, including in Bangladesh. This study analyzed a nationally representative large cross-sectional dataset from Bangladesh, and aims to contribute to understandings of the issue that will lead to appropriate strategies to overcome the challenge. There are several limitations that should be given consideration for further studies. First, it is not possible to check causality of association by cross-sectional data. Second, The World Health Organization and the Global Nutritional Community have separately set different cut-off points for BMI classification which may cause variation in categorization of an individual body mass index. Third, an asset-based proxy indicator- “wealth index” is used for understanding household economic status which does not provide unique results those obtained from income or expenditure. Fourth, due to the huge number of missing values in the dataset we had to extract only 1701 observations from the Women’s questionnaire on the basis of several selected socioeconomic indicators responsible for the prevalence of overweight and obesity. Lastly, controlling potential confounders such as energy intake, smoking, physical activity, body composition, and visceral adiposity were not addressed in the regression estimation as BDHS usually do not collect detailed health data on the above mentioned variables. The influence of any of these confounding variables might lead to inaccurate results.

## Conclusions

Our study found notable high prevalence of overweight and obesity among Bangladeshi urban women. We also found a number of socioeconomic factors for being overweight and obese among urban women of reproductive age. These factors include age, wealth index, education and marital status. This suggests that strategies and policies that place particular emphasis on older, more highly educated urban women are needed. The findings also suggest that strategies aimed at both poorer and richer women also need to be implemented. The expected cost associated with being overweight and obese is not only a burden for individual or families but for the country as a whole. The burden of overweight and obesity adversely affects labor supply and productivity, in turn reducing economic growth [45]. Further, more in-depth research including several important factors such as nutritional history, physical activity level, and central obesity related indicators are also required to overcome the challenge of overweight and obesity.

## Data Availability

The data demonstrated in this study drawn from the most recent 2014 Bangladesh Demographic Health Survey (BDHS), which is available on request at http://dhsprogram.com/what-we-do/survey/survey-display-461.cfm

## Abbreviations

BBS: Bangladesh Bureau of Statistics
BDHS: Bangladesh Demographic and Health Survey
CADs: Coronary artery diseases
DHS: Demographic and Health Survey
NCDs: Non-communicable diseases
OA: Osteoarthritis
PHC: Population and Housing Census
USAID: United States Agency for International Development

## References

[1] Cesare MD, Bentham J, Stevens GA, Zhou B, Danaei G, Lu Y, Bixby H, Cowan MJ, Riley LM, Hajifathalian K. Trends in adult body-mass index in 200 countries from 1975 to 2014: a pooled analysis of 1698 population-based measurement studies with 19.2 million participants. Lancet. 10.1016/S0140-6736(16)30054-X PMC7615134 27115820

[2] Mathers CD, Loncar D (2006). Projections of global mortality and burden of disease from 2002 to 2030. PLoS Med.

[3] Eckel RH, York DA, Rössner S, Hubbard V, Caterson I, Jeor STS, Hayman LL, Mullis RM, Blair SN (2004). Prevention conference VII: obesity, a worldwide epidemic related to heart disease and stroke: executive summary. Circulation.

[4] Kopelman P (2007). Health risks associated with overweight and obesity. Obes Rev.

[5] Obesity and overweight**.** http://www.who.int/en/news-room/fact-sheets/detail/obesity-and-overweight. Accessed 20 Nov 2017.

[6] Hu FB (2003). Overweight and obesity in women: health risks and consequences. J Womens Health.

[7] Campbell T, Campbell A (2007). Emerging disease burdens and the poor in cities of the developing world. J Urban Health.

[8] Ziraba AK, Fotso JC, Ochako R (2009). Overweight and obesity in urban Africa: a problem of the rich or the poor?. BMC Public Health.

[9] Sarma H, Saquib N, Hasan MM, Saquib J, Rahman AS, Khan JR, Uddin MJ, Cullen MR, Ahmed T (2016). Determinants of overweight or obesity among ever-married adult women in Bangladesh. BMC Obesity.

[10] Hoque ME, Long KZ, Niessen LW, Mamun AA (2015). Rapid shift toward overweight from double burden of underweight and overweight among Bangladeshi women: a systematic review and pooled analysis. Nutr Rev.

[11] Kamal SM, Hassan CH, Alam GM (2015). Dual burden of underweight and overweight among women in Bangladesh: patterns, prevalence, and sociodemographic correlates. J Health Popul Nutr.

[12] Khan MM, Krämer A (2009). Factors associated with being underweight, overweight and obese among ever-married non-pregnant urban women in Bangladesh. Singap Med J.

[13] Shafique S, Akhter N, Stallkamp G, de Pee S, Panagides D, Bloem MW (2007). Trends of under-and overweight among rural and urban poor women indicate the double burden of malnutrition in Bangladesh. Int J Epidemiol.

[14] The DHS Program. Demographic and Health Surveys. https://dhsprogram.com/. Accessed 10 Jan 2017.

[15] Bangladesh Urban Health Survey 2013. Final Report. https://www.measureevaluation.org/resources/publications/tr-15-117. Accessed 10 Jan 2017.

[16] Oddo VM, Rah JH, Semba RD, Sun K, Akhter N, Sari M, de Pee S, Moench-Pfanner R, Bloem M, Kraemer K (2012). Predictors of maternal and child double burden of malnutrition in rural Indonesia and Bangladesh. Am J Clin Nutr.

[17] Neupane S, Prakash K, Doku DT (2015). Overweight and obesity among women: analysis of demographic and health survey data from 32 sub-Saharan African countries. BMC Public Health.

[18] Popkin BM, Gordon-Larsen P (2004). The nutrition transition: worldwide obesity dynamics and their determinants. Int J Obes.

[19] Keding GB, Msuya JM, Maass BL, Krawinkel MB (2013). Obesity as a public health problem among adult women in rural Tanzania. Glob Health Sci Pract.

[20] Ridker PM, Manson JE, Ridker PM, Gaziano JM, Hennekens CH (1996). The pathogenesis of atherosclerosis and acute thrombosis: relevance to strategies of cardiovascular disease prevention. Prevention of Myocardial Infarction.

[21] Janghorbani M, Amini M, Willett WC, Gouya MM, Delavari A, Alikhani S, Mahdavi A (2007). First nationwide survey of prevalence of overweight, underweight, and abdominal obesity in Iranian adults. Obesity.

[22] Bangladesh Bureau of Statistics. StatisticalYearBookFinal2016**.** bbs.portal.gov.bd/sites/default/files/files/bbs…/StatisticalYearBookFinal2016.pdf*.* Accessed 30 Feb 2018.

[23] United Nations Development Programme**.** Human development report 2016**.** http://hdr.undp.org/sites/default/files/2016_human_development_report.pdf*.* Accessed 3 Mar 2018.

[24] United Nations. World Urbanization Prospects. 2014. ISBN: 978-92-1-151517-6.

[25] Bangladesh Demographic and Health Survey 2014. https://dhsprogram.com/pubs/pdf/FR311/FR311.pdf. Accessed 14 Oct 2016.

[26] World Health Organization (2000). Obesity: preventing and managing the global epidemic: report of a WHO consultation (WHO technical report series 894).

[27] Abrha S, Shiferaw S, Ahmed KY (2016). Overweight and obesity and its socio-demographic correlates among urban Ethiopian women: evidence from the 2011 EDHS. BMC Public Health.

[28] Kandala N-B, Stranges S (2014). Geographic variation of overweight and obesity among women in Nigeria: a case for nutritional transition in sub-Saharan Africa. PLoS One.

[29] Puoane T, Steyn K, Bradshaw D, Laubscher R, Fourie J, Lambert V, Mbananga N (2002). Obesity in South Africa: the south African demographic and health survey. Obesity.

[30] Gbary AR, Kpozehouen A, Houehanou YC, Djrolo F, Amoussou MP, Tchabi Y, Salamon R, Houinato DS (2014). Prevalence and risk factors of overweight and obesity: findings from a cross-sectional community-based survey in Benin. Glob Epidemic Obes.

[31] Kaur G, Singh S, Singh A (2013). Prevalence of overweight and obesity in urban and rural women of Punjab. Hum Bio Rev.

[32] Mendez MA, Monteiro CA, Popkin BM (2005). Overweight exceeds underweight among women in most developing countries. Am J Clin Nutr.

[33] Doak CM, Adair LS, Monteiro C, Popkin BM (2000). Overweight and underweight coexist within households in Brazil, China and Russia. J Nutr.

[34] Caballero B (2007). The global epidemic of obesity: an overview. Epidemiol Rev.

[35] Joshi P, Islam S, Pais P, Reddy S, Dorairaj P, Kazmi K, Pandey MR, Haque S, Mendis S, Rangarajan S (2007). Risk factors for early myocardial infarction in south Asians compared with individuals in other countries. Jama.

[36] Subramanian SV, Perkins JM, Khan KT (2009). Do burdens of underweight and overweight coexist among lower socioeconomic groups in India?. Am J Clin Nutr.

[37] Alemu E, Atnafu A, Yitayal M, Yimam K (2014). Prevalence of overweight and/or obesity and associated factors among high school adolescents in Arada sub city, Addis Ababa, Ethiopia. J Nutr Food Sci.

[38] Wells JC, Siervo M (2011). Obesity and energy balance: is the tail wagging the dog?. Eur J Clin Nutr.

[39] Gallagher D, Visser M, De Meersman RE, Sepúlveda D, Baumgartner RN, Pierson RN, Harris T, Heymsfield SB (1997). Appendicular skeletal muscle mass: effects of age, gender, and ethnicity. J Appl Physiol.

[40] Villareal DT, Apovian CM, Kushner RF, Klein S (2005). Obesity in older adults: technical review and position statement of the American Society for Nutrition and NAASO, the Obesity Society. Obesity.

[41] Du S, Mroz TA, Zhai F, Popkin BM (2004). Rapid income growth adversely affects diet quality in China—particularly for the poor!. Soc Sci Med.

[42] Wamala SP, Wolk A, Orth-Gomér K (1997). Determinants of obesity in relation to socioeconomic status among middle-aged Swedish women. Prev Med.

[43] Martorell R, Khan LK, Hughes ML, Grummer-Strawn LM (2000). Obesity in women from developing countries. Eur J Clin Nutr.

[44] Dastgiri S, Mahdavi R, TuTunchi H, Faramarzi E (2006). Prevalence of obesity, food choices and socio-economic status: a cross-sectional study in the north-west of Iran. Public Health Nutr.

[45] Greve J (2008). Obesity and labor market outcomes in Denmark. Econ Hum Biol.

